# Safety Study of an Antimicrobial Peptide Lactocin 160, Produced by the Vaginal *Lactobacillus rhamnosus*

**DOI:** 10.1155/2007/78248

**Published:** 2007-12-09

**Authors:** Sara E. Dover, Alla A. Aroutcheva, S. Faro, Michael L. Chikindas

**Affiliations:** ^1^School of Biotechnology, The Royal Institute of Technology, 100 44 Stockholm, Sweden; ^2^Rutgers, The State University of New Jersey, New Brunswick, NJ 08901-8520, USA; ^3^Rush Medical Center, Chicago, IL 60612, USA; ^4^Health Promoting Naturals, Inc., Highland Park, NJ 08904, USA; ^5^The Women's Hospital of Texas, Houston, TX 77054, USA

## Abstract

*Objective.* To evaluate the safety of the antimicrobial peptide, lactocin 160. *Methods.* Lactocin 160, a product of vaginal probiotic *Lactobacillus rhamnosus* 160 was evaluated for toxicity and irritation. An in vitro human organotypic vaginal-ectocervical tissue model (EpiVaginal) was employed for the safety testing by determining the exposure time to reduce tissue viability to 50% (ET-50). Hemolytic activity of lactocin160 was tested using 8% of human erythrocyte suspension. Susceptibility of lactobacilli to lactocin160 was also studied. Rabbit vaginal irritation (RVI) model was used for an in vivo safety evaluation. *Results.* The ET-50 value was 17.5 hours for lactocin 160 (4.9 hours for nonoxynol 9, N9). Hemolytic activity of lactocin 160 was 8.2% (N9 caused total hemolysis). Lactobacilli resisted to high concentrations of peptide preparation. The RVI model revealed slight vaginal irritation. An average irritation index grade was evaluated as “none.” *Conclusions.* Lactocin 160 showed minimal irritation and has a good potential for intravaginal application.

## 1. INTRODUCTION

*Lactobacillus rhamnosus* 160 was isolated from the vaginal microflora of a healthy female subject. This bacterium produces the ribosomally synthesized antimicrobial peptide (bacteriocin) lactocin 160, which is active against the most prevalent species associated with bacterial vaginosis (BV). Its bactericidal activity (as shown on model microorganisms) is due to the disturbance of the cellular membrane, probably by pore formation, which leads to efflux of ATP [1, 2].

BV is a common condition in women which occurs when the healthy vaginal bacterial flora, consisting mostly of *Lactobacillus* species, is replaced by a flora consisting of several bacterial pathogens [3]. It is associated with pregnancy complications such as premature labor as well as with a higher risk of acquisition of HIV infection [4–6].

Since lactocin 160 is active against an array of vaginal bacterial pathogens, it has a potential application as an antimicrobial treatment of BV. It is, therefore, important to determine the safety of the bacteriocin for human application. For in vitro toxicity study, the EpiVaginal tissue model from MatTek Corporation (Ashland, Mass, USA) was employed. This model uses human vaginal ectocervical cells, obtained from healthy adult females, and is free from viral, microbial, and yeast infections. The tissue has a three-dimensional structure that mimics that of the human vaginal tissue and the results are highly reproducible, quantifiable, and less expensive compared to in vivo studies [7]. The in vitro data was compared with rabbit vaginal irritation (RVI) model. The aim of this study was an in vitro and in vivo testing of antimicrobial peptide lactocin 160 to determine vaginal tissue toxicity, hemolytic activity, and inhibition of lactobacilli growth.

## 2. METHODS

### 2.1. Production of lactocin 160

*Lactobacillus rhamnosus* strain 160 was stored in the biofreezer (−80°C) until use. Lactocin 160 was purified as described previously [1]. Briefly, *L*. *rhamnosus* 160 was grown anaerobically overnight in 2000 mL MRS (Difco Lactobacilli MRS broth) at $37^{{}^{\circ}}$C. The cells were harvested by centrifugation (5100 x*g*, 20 minutes, 4°C), and washed three times in 0.01 M phosphate buffered saline (PBS, Sigma-Aldrich, Mo, USA). The pellet was resuspended in 200 mL chemically defined media that resembles vaginal fluid but lacks proteins [8], and incubated anaerobically at 37°C for 20 hours with agitation (100 rpm).

The supernatant was brought to 80% saturation with ammonium sulphate (Sigma-Aldrich). Precipitated (not active) proteins were removed by centrifugation (11,000 x*g*, 25 minutes, 4°C). The supernatant was dialyzed against double distilled water for three days with the molecular weight cutoff of 1 kDa, (Spectra/Por Cellulose ester dialysis membranes, Spectrum). This was followed by lyophilization to obtain dry lactocin 160 preparation.

### 2.2. Protein concentration

Lactocin 160 is a partially purified protein preparation and contains other high molecular products, with trace amounts of salts from the medium. Protein concentration was measured using BCA method (Pierce, Ill, USA) and determined spectrophotometrically at 562 nm.

### 2.3. Determination of weak organic acid presence in the preparation

Prior to the toxicity study, lactocin 160 was resuspended in double distilled water at a concentration of 200 mg/mL. The samples’ acidity was analyzed by potentiometer (Microcomputer pH-vision 6071, Markson, Honolulu, Hawaii, USA) and the lactic acid concentration was measured using a commercial D- and L-lactic acid testing kit (R-Biopharm AG, Darmstadt, Germany). The sample was filter sterilized using a 0.45 μm centrifuge filter (Millipore Corporation, Billerica, Mass, USA).

### 2.4. HPLC and electrospray-MS analysis

To determine reproducibility of the composition of the partially purified lactocin 160 preparations, samples from 3 independent batches were submitted to Proteomics Resource Facility (Integrated Biotechnology Laboratories, University of Georgia, Ga, USA) for HPLC and electrospray-MS analysis.

### 2.5. Epivaginal tissues model

EpiVaginal tissues (VEC-100) were obtained from MatTek Corporation and placed in the refrigerator at 4°C until use. All tissues were used within 24 hours. Prior to application of the product, tissues were preequilibrated in 6 well plates (Falcon) with 0.9 mL of DMEM-based DC-100 MM medium (MatTek Corporation). Plates containing tissue culture inserts were placed in a humidified incubator at 37°C and 5% CO_2_ for one hour. Media was removed and replaced by 0.9 mL of fresh VEC-100-MM medium. Then, 83 μL of product was applied topically on triplicate EpiVaginal (VEC-100) tissues. For exposure times of greater than 24 hours, tissue inserts were airlifted by placing them on 2 washers (MatTek Corporation) and fed with 5 mL of the assay medium. Initial exposure times of lactocin 160 (200 mg/mL) were 4, 9, 24, and 48 hours. Distilled water, used as negative control, was applied to duplicate tissue culture inserts at time points 6, 24, and 48 hours. Spermicide containing 4% Nonoxynol-9 (Ortho Options CONCEPTROL Vaginal Contraceptive Gel, Advanced Care Products, Skillman, NJ, USA) has a well-documented cytotoxicity (7, 9, 10), and was therefore chosen as a positive control. Antifungal cream, containing 4% miconazole nitrate (Monistat-3, Ortho McNeil Pharmaceutical, Inc., Raritan, NJ, USA), is a nontoxic preparation and was used as a negative control [7, 9–11].

Tissue viability at the end of the exposure time was determined using the MTT (3-(4,5-Dimethylthiazol-2-yl)-2,5-diphenyltetrazolium bromide) viability assay. Approximate effective times (ET) for each product to reduce tissue viability to 50% (ET-50) values were calculated, from which new time points were selected to obtain a more accurate ET-50 value.

### 2.6. MTT viability assay

Viability of cells was determined by measuring the breakdown of the yellow tetrazolium component MTT to purple formazan following manufacturers’ (MatTek Corporation) recommendations. As only living cells perform breakdown of MTT, the reduction is directly proportional to the amount of living cells [12]. Briefly, after completion of exposure times of lactocin 160 and positive and negative controls to tissues, the liquid in the inserts was decanted, followed by washing the tissue inserts in Dulbeccos phosphate-buffered saline solution (D-PBS). Nonliquid test material was removed carefully with a sterile polyester fiber tipped swab (Thermo- Fisher, Waltham, Mass, USA). Tissues were then placed in a 24 well plate with 300 μL MTT (1.0 mg/mL) solution in culture medium and incubated for 3 hours at 37°C and 5% CO_2_. Following MTT incubation, the tissue inserts were placed in a new 24 well plate and immersed in 1.66 mL of isopropanol (MatTek Corporation) to extract the formazan. To reduce extractant evaporation, the plate was covered with sealing film (Thermo-Fisher). The plate was incubated overnight in the dark at room temperature. Upon completion of extraction, the liquid from the wells was mixed with the liquid from the inserts. After mixing, 200 μL of extractant solution was measured spectrophotometrically in triplicate at 570 nm in a 96 well plate reader (MRX revelation, Dynex Technologies, Va, USA) using extractant solution as blank.

Tissue viability (%) was determined according to the manufacturer’s recommendations and using the equation: $\%\text{viability}={\text{OD}}_{570}(\text{treated}\;\text{tissue})/{\text{OD}}_{570}(\text{control}\;\text{tissue})$. The exposure time that reduced the tissue viability to 50% was calculated as described previously [7] by plotting the logarithm of the dosing time versus % viability and then interpolating the times near when the viability is 50%. In general, a shorter ET-50 corresponds to a more irritating/damaging test article; a longer ET-50 corresponds to milder/less damaging test article.

### 2.7. Reduction of MTT by test preparations

In order to assure reduction of MTT was not caused by the test articles, thereby generating incorrect results, reduction of MTT by each product was measured. This was done by adding 83 μL of product to 1 mL of MTT solution and incubating at room temperature in the dark for 60 minutes. 83 μL of double distilled water in 1 mL of MTT solution was used as negative control. Absence of darkening of solution color indicated that the test article and controls did not reduce MTT.

### 2.8. Rabbit vaginal model

This safety assay was conducted at Eurofins Product Safety Laboratories (Dayton, NJ, USA). This study was performed to comply with the Good Laboratory Practice (GLP) regulations as defined in: 21 CFR 58: U.S. FDA Good Laboratory Practice Standards.

A quantity equal to 1.8 mL of the test article at pH 6.0, containing 10 mg/mL dissolved in sterile distilled water was administered intravaginally to six healthy female rabbits. One dose was administered daily to each animal for ten consecutive days. A negative and positive control group, consisting of six female rabbits each, was maintained under the same environmental conditions and dosed daily with 1.8 mL of sterile distilled water and 4% nonoxynol-9, respectively. All animals were observed for mortality, signs of gross toxicity, and behavioral changes at least once daily for 11 days. Body weights were recorded prior to administration and on day 11 (study termination). Individual vaginal irritation was measured prior to each intravaginal administration and on day 11. The entire vagina was excised, examined, and scored for exudates (vaginal discharge); edema; and erythema. The vagina from each rabbit was fixed and saved in 10% neutral buffered formalin. Scores for the vaginal discharge and edema were: 0—normal/none, 1—slight, 2—moderate, 3—severe, and scores for erythema were: 0—normal, 1—pink, 2—red, 3—beet red. Necropsies were performed on all animals at terminal sacrifice. Three sections of the columnar epithelium of the vagina (including cervical, central, and caudal portions) were examined microscopically from hematoxylin- and eosin-stained slides. The maximum score for the microscopic evaluation was 16.

### 2.9. Hemolysis assay

Outdated human blood erythrocytes obtained from the Blood Center of the Rush University of Chicago (Ill, USA) were used for the determination of hemolysis. Serial dilutions of lactocin 160 (12.5–200 mg/mL) were prepared in PBS. 100 μL aliquots of each dilution were incubated with 1 mL of 8% erythrocyte suspension in 1x PBS for 15 minutes at 22°C, and then centrifuged at 1500 rpm for 1 minute. The absorbance at 405 nm of released hemoglobin was measured spectrophotometrically. Hemolytic activities are presented as the percentage of the total erythrocytes lysed [13]. Sodium dodecyl sulfate was used as a control for total lysis. Hemolytic activity of lactocin160 was compared to 1–4% N9.

### 2.10. Inhibition of vaginal lactobacillus

The inhibitory effect of lactocin 160 on bacterial species characteristic of a healthy vaginal flora was studied against 10 vaginal *Lactobacillus* species (healthy human subjects isolates) by determining minimum inhibitory concentration (MIC) using the microdilution method in MRS broth. The tested range of two-fold dilution of lactocin 160 was 1.5–200 mg/mL with inoculum size of $5\times 10^{5}$ CFU/mL. Loaded wells were overlaid with sterile mineral oil to create anaerobic condition for lactobacilli growth. MIC was determined using Bioscreen reader-incubator (Labsystems, Helsinki, Finland).

## 3. RESULTS

### 3.1. Lactocin 160 preparation analysis

An amount of 100 mg of partially purified lactocin 160 contained 21.5±1.29 μg protein. The test for lactic acid detected less than 1% L-lactic acid concentration of the dry weight of three samples of lactocin160; D-lactic acid was found in insignificant amounts. This test confirmed that the activity of lactocin160 was not caused by lactic acid.

### 3.2. HPLC and electrospray-MS analysis

The HPLC analysis was used to confirm reproducibility of the peptide recovery method from three independent batches of lactocin 160. It revealed that the samples have very similar protein profiles (graphs not shown). The electrospray-MS analysis confirmed the reproducibility data with significant noise due to the presence of amino acids (data not shown).

### 3.3. ET-50 values

**Figure 1:**
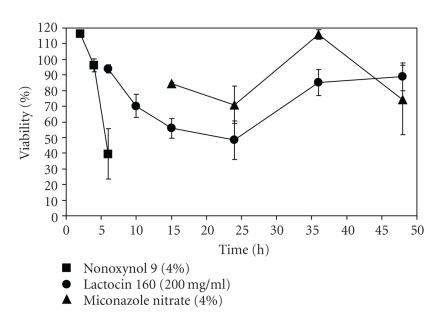
Lactocin 160 preparation is not toxic for human vaginal cells as tested in EpiVaginal tissue (VEC-100) model. The presented data illustrate %viability versus time (hours) for following preparations: ▪, nonoxynol 9 (4%); •, lactocin 160 (200 mg/mL); ▴, miconazole nitrate (4%).

The concentration of 200 mg/mL of lactocin 160 preparation was chosen for the in vitro safety study as this higher concentration is more likely to later be used in a commercial product. Lactocin 160 at this concentration showed very little irritation, with an average ET-50 value of 17.5 hours. Table 1 summarizes the ET-50 values for the samples. As an example, Figure 1 presents the data from one study.

As expected, N9 was most toxic for EpiVaginal cells, with an average ET-50 value of 4.9 hours. This confirms previous in vitro and in vivo studies [7, 14, 15]. With an ET-50 value of >24 hours, miconazole nitrate showed very low toxicity in our study. This is in accordance with previous in vitro and in vivo testing using the RVI model and clinical trials [7, 9–11].

An increase in liquid was observed in the insert of tissue exposed to lactocin 160. This might have been due to differences between products and media, thereby causing water to enter the tissue insert by osmotic pressure. It may also be due to bacteriocins inducing EpiVaginal cells to produce mucin. As the liquid in the wells was not viscous, the first speculation is more likely to have account for the observation (personal communication with Dr. Ayehunie at MatTek Corp.).

### 3.4. Reduction of MTT by test article

MTT was not reduced to formazan by lactocin 160, micronazole nitrate, or nonoxynol 9. Thus, reduction of MTT was only performed by viable cells, and not caused by test article. This confirms the validity of the assay.

### 3.5. Rabbit safety assay

All animals survived, gained body weight, and appeared active and healthy during the study. In control group with 4% N9 apart from a red gelatinous substance in the panline noted for one animal on day 2, there were no other signs of gross toxicity, adverse pharmacologic effects, or abnormal behavior (Table 3). Slight-to-moderate vaginal discharge and pink-to-red erythema were noted for all treated sites between days 3 and 11. No gross abnormalities were noted for any of the animals when necropsied at the conclusion of the 11-day observation period. Slight vaginal discharge, pink-to-red erythema, and/or slight-to-moderate edema were observed in all six rabbits at time of necropsy. The positive for irritation control tissues (nonoxynol-9 treatment) were given an average irritation index grade of “mild.”

In the test group (lactocin 160 treatment), slight vaginal discharge and/or pink erythema were noted for three treated sites between days 5 and 11. No gross abnormalities were noted for any of the animals when necropsied at the conclusion of the 11-day observation period. Slight-to-moderate vaginal discharge, pink-to-red erythema, and/or slight-to-moderate edema were observed in all six rabbits at time of necropsy. The test tissues for lactocin 160 were given an average irritation index grade of “none.” Under the conditions of this test, the lactocin 160 did not produce any significant vaginal irritation.

### 3.6. Toxicity for lactobacilli

Lactocin 160 preparation’s MIC for the tested 10 vaginal *Lactobacillus* spp was >200 mg/mL.

### 3.7. Hemolytic activity

Table 3 presents data of human erythrocyte hemolysis by lactocin 160 (12.5–200 mg/mL) and N9. The range of observed hemolysis was 1.2±0.34–8.2±0.6%. In fact, N9 in concentration 1–4% caused total hemolysis of erythrocytes.

## 4. DISCUSSION

Lactocin 160, an antimicrobial peptide produced by *Lactobacillus rhamnosus*, did not show any severe irritation in the RVI model, ectovaginal tissue, hemolytic activity, or vaginal lactobacilli inhibition. The ET-50 values of the lactocin 160 preparations were similar or less than over-the-counter nontoxic products. Our toxicity studies revealed that lactocin 160 can be considered safe for human use.

For vaginal toxicity studies, thein vivo RVI model has been the preferred choice for a long time. This model determines the cervicovaginal irritation as minimal, mild, moderate, and severe based on a scoring system evaluating epithelial ulceration, leucocytic infiltration, edema, and vascular injection [16]. As it is an established standard, the results can be compared to other products [17–19]. The RVI model is, however, best for identifying agents that will cause severe irritation [20]. This model poses some shortcomings, mainly because of the difference in vaginal tissue between rabbit and human. Also, extra variables have to be taken into account, for example, the physical status, the health history of the rabbit, and the immune system. The RVI model approach proved not to be totally reliable in the case of the spermicidal agent nonoxynol 9. This commonly used contraceptive was determined to be suitable for human use in the rabbit irritation model; but with frequent use it induces inflammatory reactions and disruption of the vaginal epithelia [14, 15]. This has possible adverse effects including higher risk of acquisition of HIV [21]. In addition, prolonged exposure (10 days in different concentrations) to N9 in the rabbit vagina revealed severe inflammatory changes including erosions and loss of epithelial lining. The damage was proportional to the amount of N9 used [22].

In vitro models for vaginal irritation include monolayers of cells, which have been used for many years to determine safety, for example, endocervical vaginal epithelial HeLa-S3 cells [23] and immortalized human vaginal and cervical epithelial cells [24–26]. However, monolayer cultures are not optimal for irritation studies as they do not accurately resemble three-dimensional, differentiated vaginal tissue and also have different gene and protein expression [7].

Three-dimensional models of normal ectocervicovaginal epithelial cells, grown on polycarbonate filters, have previously been used. In a study of the pokeweed antiviral protein microbicide, no correlation was found between the results of the in vitro and the RVI model, the latter being much more sensitive [18]. These researchers hypothesized that this might be due to the in vitro system lacking the dynamics of in vivo testing. The inflammation may have been caused by the product’s effect on mast cells, thereby creating the inflammatory reaction instead of a direct cytotoxic effect to the vaginal epithelial cells.

In this study, we sought to transition from the traditional in vivo vaginal irritation test to a reliable and reproducible in vitro vaginal model. We chose EpiVaginal model because it is a three-dimensional organotypic ectocervicovaginal tissue that was reconstructed using normal, well-stratified epithelial cells, containing differentiated basal, suprabasal, intermediate, and superficial cell layers similar to in vivo tissue. The in vivo and in vitro results were similar and revealed little or no toxicity of lactocin 160. This in vitromodel is cost-effective and can be considered as an alternative to the animal model.

To the best of our knowledge, this is the first in vitro vaginal irritation study of a bacteriocin from a vaginal probiotic *Lactobacillus* strain using EpiVaginal tissues. Vaginal safety was studied for the bacteriocin nisin. This well-known bacteriocin is widely used for food preservation but is not produced by any vaginal isolates [23, 27]. Nisin has been proposed as a contraceptive agent even though it kills the healthy vaginal microflora consisting of *Lactobacillus* species in concentrations much lower than the ones proposed for the nisin’s spermicidal application (our unpublished data). The absence of vaginal irritation of nisin in rat and rabbit model was confirmed in vitro using endocervical vaginal epithelial HeLa-S3 cells measuring viability [23, 28]. Also, nisin’s hemolytic activity has been studied well. It was found that nisin caused hemolysis of sheep erythrocytes at concentrations that were 1000-fold higher than those required for antimicrobial activity [29].

Documenting the antimicrobial activity against vaginal lactobacilli is an important concern for vaginal formulation safety study. For instance, it was shown that presence of nonoxinol-9 affects the ecological balance of the vagina by inhibiting the protector lactobacilli [30–32]. At the same time, our in vitro and in vivo toxicity studies showed that the antimicrobial peptide lactocin 160 produced by vaginal *L. rhamnosus* did not irritate vaginal epithelial tissue, and it was not hemolytic for human erythrocytes or toxic for vaginal lactobacilli. These results suggest that it would be safe in formulations to treat bacterial vaginosis.

## Figures and Tables

**Table 1:** ET-50 values of products using the EpiVaginal tissue model (mean± standard deviation).

Product and active ingredient	ET-50 (h)
Lactocin 160 (200 mg/mL)	17.45±0.35
Spermicide	
Nonoxynol 9 (4%)	4.85±1.06
Antifungal cream	
Miconazole nitrate (4%)	35.6±17.5

**Table 2:** Individual vaginal irritation and histopathology scores. Histopathology scores include evaluation of epithelium, leukocytes infiltration, vascular congestion, and edema.

Average score	Vaginal discharge/erythema										Histopathology score (Group mean)

Day	2	3	4	5	6	7	8	9	10	11	11
Distill water (n=6)	0.0/0.0	0.0/0.0	0.0/0.0	0.0/0.0	0.0/0.0	0.0/0.0	0.0/0.0	0.0/0.0	0.0/0.0	0.0/0.0	5.2
N-9 (n=6)	0.0/0.0	0.3/0.5	0.3/0.5	0.3/0.7	0.3/0.7	0.8/1.0	1.2/1.0	1.0/1.3	1.7/1.5	1.3/1.3	10.5
Lactocin 160 (n=6)	0.0/0.0	0.0/0.0	0.0/0.0	0.0/0.2	0.0/0.2	0.0/0.2	0.3/0.3	0.3/0.3	0.3/0.3	0.3/0.3	5.7

**Table 3:** Hemolytic activity of lactocin 160.

Substance	Lactocin 160 (mg/mL)					N9 (%)
Concentration	200	100	50	25	12.5	1–4

Hemolytic activity (% of control)	8.2±0.6	6.2±0.2	4.1±0.28	1.1±0.34	0	100

## References

[1] Aroutcheva AA, Simoes JA, Faro S (2001). Antimicrobial protein produced by Vaginal *Lactobacillus acidophilus* that inhibits *Gardnerella vaginalis*. *Infectious Diseases in Obstetrics and Gynecology*.

[2] Li J, Aroutcheva AA, Faro S, Chikindas ML (2005). Mode of action of lactocin 160, a bacteriocin from vaginal *Lactobacillus rhamnosus*. *Infectious Diseases in Obstetrics and Gynecology*.

[3] Hill GB (1993). The microbiology of bacterial vaginosis. *American Journal of Obstetrics and Gynecology*.

[4] Hillier SL, Nugent RP, Eschenbach DA (1995). Association between bacterial vaginosis and preterm delivery of a low-birth-weight infant. *The New England Journal of Medicine*.

[5] Flynn CA, Helwig AL, Meurer LN (1999). Bacterial vaginosis in pregnancy and the risk of prematurity: a meta-analysis. *The Journal of Family Practice*.

[6] Martin HL, Richardson BA, Nyange PM (1999). Vaginal lactobacilli, microbial flora, and risk of human immunodeficiency virus type 1 and sexually
transmitted disease acquisition. *Journal of Infectious Diseases*.

[7] Ayehunie S, Cannon C, Lamore S (2006). Organotypic human vaginal-ectocervical tissue model for irritation studies of spermicides, microbicides, and
feminine-care products. *Toxicology in Vitro*.

[8] Geshnizgani AM, Onderdonk AB (1992). Defined medium simulating genital tract secretions for growth of vaginal microflora. *Journal of Clinical Microbiology*.

[9] Davis JE, Frudenfeld JH, Goddard JL (1974). Comparative evaluation of Monistat and Mycostatin in the treatment of vulvovaginal candidiasis. *Obstetrics & Gynecology*.

[10] Sawyer PR, Brogden RN, Pinder RM, Speight TM, Avery GS (1975). Miconazole: a review of its antifungal activity and therapeutic efficacy. *Drugs*.

[11] Ozyurt E, Toykuliyeva MB, Danilyans IL, Morton O, Baktir G (2001). Efficacy of 7-day treatment with metronidazole+miconazole (Neo-Penotran®)-a triple-active pessary
for the treatment of single and mixed vaginal infections. *International Journal of Gynecology & Obstetrics*.

[12] Mosmann T (1983). Rapid colorimetric assay for cellular growth and survival: application to proliferation and cytotoxicity assays. *Journal of Immunological Methods*.

[13] Hertle R, Brutsche S, Groeger W (1997). Specific phosphatidylethanolamine dependence of *Serratia marcescens* cytotoxin activity. *Molecular Microbiology*.

[14] Niruthisard S, Roddy RE, Chutivongse S (1991). The effects of frequent nonoxynol-9 use on the vaginal and cervical mucosa. *Sexually Transmitted Diseases*.

[15] Hoffman IF, Taha TE, Padian NS (2004). Nonoxynol-9 100 mg gel: multi-site safety study from sub-Saharan Africa. *AIDS*.

[16] Eckstein P, Jackson MC, Millman N, Sobrero AJ (1969). Comparison of vaginal tolerance tests of spermicidal preparations in rabbits and monkeys. *Journal of Reproduction and Fertility*.

[17] Zaneveld LJ, Waller DP, Anderson RA (2002). Efficacy and safety of a new vaginal contraceptive antimicrobial formulation containing high
molecular weight poly(sodium 4-styrenesulfonate). *Biology of Reproduction*.

[18] D'Cruz OJ, Waurzyniak B, Uckun FM (2004). Mucosal toxicity studies of a gel formulation of native pokeweed antiviral protein. *Toxicologic Pathology*.

[19] Dhondt MM, Adriaens E, Roey JV, Remon JP (2005). The evaluation of the local tolerance of vaginal formulations containing dapivirine using the Slug
Mucosal Irritation test and the rabbit vaginal irritation test. *European Journal of Pharmaceutics and Biopharmaceutics*.

[20] Doncel GF, Chandra N, Fichorova RN (2004). Preclinical assessment of the proinflammatory potential of microbicide candidates. *Journal of Acquired Immune Deficiency Syndromes*.

[21] Van Damme L, Ramjee G, Alary M (2002). Effectiveness of COL-1492, a nonoxynol-9 vaginal gel, on HIV-1 transmission in female sex workers:
a randomised controlled trial. *The Lancet*.

[22] Chvapil M, Droegemueller W, Owen JA, Eskelson CD, Betts K (1980). Studies of nonoxynol-9. I. The effect on the vaginas of rabbits and rats. *Fertility and Sterility*.

[23] Reddy KVR, Aranha C, Gupta SM, Yedery RD (2004). Evaluation of antimicrobial peptide nisin as a safe vaginal contraceptive agent in rabbits: in vitro and in vivo studies. *Reproduction*.

[24] Krebs FC, Miller SR, Catalone BJ (2000). Sodium dodecyl sulfate and C31G as microbicidal alternatives to nonoxynol 9:
comparative sensitivity of primary human vaginal keratinocytes. *Antimicrobial Agents and Chemotherapy*.

[25] Krebs FC, Miller SR, Catalone BJ (2002). Comparative in vitro sensitivities of human immune cell lines, vaginal and cervical epithelial cell lines,
and primary cells to candidate microbicides nonoxynol 9, C31G, and sodium dodecyl sulfate. *Antimicrobial Agents and Chemotherapy*.

[26] Fichorova RN, Bajpai M, Chandra N (2004). Interleukin (IL)-1, IL-6 and IL-8 predict mucosal toxicity of vaginal microbicidal contraceptives. *Biology of Reproduction*.

[27] Cleveland J, Montville TJ, Nes IF, Chikindas ML (2001). Bacteriocins: safe, natural antimicrobials for food preservation. *International Journal of Food Microbiology*.

[28] Aranha C, Gupta S, Reddy KVR (2004). Contraceptive efficacy of antimicrobial peptide Nisin: in vitro and in vivo studies. *Contraception*.

[29] Maher S, McClean S (2006). Investigation of the cytotoxicity of eukaryotic and prokaryotic antimicrobial peptides in intestinal epithelial cells in vitro. *Biochemical Pharmacology*.

[30] McGroarty JA, Tomeczek L, Pond DG, Reid G, Bruce AW (1992). Hydrogen peroxide production by *Lactobacillus* species: correlation with susceptibility to
the spermicidal compound nonoxynol-9. *Journal of Infectious Diseases*.

[31] Stafford MK, Ward H, Flanagan A (1998). Safety study of nonoxynol-9 as a vaginal microbicide: evidence of adverse effects. *Journal of Acquired Immune Deficiency Syndromes and Human Retrovirology*.

[32] Pascual LM, Daniele MB, Pájaro C, Barberis L (2006). *Lactobacillus* species isolated from the vagina: identification, hydrogen peroxide production and
nonoxynol-9 resistance. *Contraception*.

